# Books

**Published:** 1882-07

**Authors:** 


### Books.

A Hand-Book of Uterine Therapeutics and Diseases of Women. By Edward John Tilt, M.D. Fourth Edition. New York: William Wood & Co. 1881. Wood's Library of Standard Medical Authors.

This book is the November, 1881, number of Wood's library, and the delay in its reception has caused the lateness of this notice. The last English edition was published in 1878. The writer thinks that the therapeutical teachings of the present day may be grouped into three classes, each contending for the mastery. One trusts in medicine alone and deprecates resort to surgical procedures. Another puts little or no trust in medicines, and adopts the knife as the safe and certain means of cure, while orthopedy is the confident resource of the third class.

"In all that I have published," says the writer, "during the last thirty years, it has been my earnest desire to combat these exaggerations by determining the proper scope and relative value of drugs, of the knife, and of pessaries in the treatment of disease;" and in this work the guiding principles which he enunciates are the importance of female hygiene; the constitutional origin of many of the diseases of women, and the necessity, therefore, of combatting them by the administration of general remedies; the possibility of curing most of the diseases of women without resort to surgery; and the impossibility of relieving some uterine disorders without the aid of surgery.

Following this line of practice, it can easily be conceived that the views and doctrines laid down in this volume are broad and conservative. Nothing especially novel or startling is presented, but generally accepted and established opinions are placed before the reader in a carefully systematized and agreeable form, and while the author is naturally intensely English in his sympathies and associations, he does not ignore American authorities.

Dr. Marion Sims is frequently referred to, and Dr. Henry F. Campbell and Dr. Robert Battey both receive attention. The former in connection with the genu pectoral position which is warmly commended, and the latter in connection with ovariotomy; though Battey's operation is not discussed, and no opinion is expressed for or against it as a legitimate surgical procedure.

In the chapter on modes of examination we find Sims' speculum denominated a *levator perinei* speculum, and the method of its use is described, accompanied by the following comment:

"This mode of examination is admirable to elucidate a recondite case, and for the performance of delicate operations on the womb and the vagina; but the profession in America employ it in all cases of uterine surgery, which is like using a steam hammer to kill a fly."

To the volume, is appended a page devoted to "investiganda," a formulary and a fair index.

---

A CLINICAL HAND-BOOK ON THE DISEASE OF WOMEN. By W. Symington Brown, M.D. New York: William Wood & Co. 1882. Pp. 246.

"Of the making of books there is no end," is the declaration of a fact that receives abundant confirmation as the years roll round. We are sometimes led to speculate upon the possible motives which prompt many writers to launch their untried barks upon the mighty and uncertain sea of literature. We shall not undertake to analyze the reasons that have actuated the author of the present volume, which he tells us does not claim to be a treatise, but "is intended as a practical guide on most of the diseases peculiar to women, for the use of students and country practitioners." He does not say wherein the needs of the country practitioner are peculiar, neither does he inform the public why he arbitrarily correlates the requirements of "medical students and country practitioners," but he nevertheless seems to have discovered a real necessity for a "practical guide on most of the diseases peculiar to women"—among which, strangely enough, we find included hemorrhoids, anal fissure, rectal polypus, rectocele, gonorrhœa, gonorrhœal rheumatism, syphilis, chanchroid and cancer—and he proceeds to supply the want. We are not quite so confident as the writer clearly is, that the demand for such a work obtains, but we can truthfully say that the book is pleasantly written, the subjects discussed are plainly and concisely presented and the doctrines which it inculcates, measured by the generally accepted views of the day, are sound.

Two pages are dedicated to "Battey's operation" which is described mainly in the words of the originator thereof, unaccompanied by comment or criticism.

The book, as a whole, deserves a friendly reception at the hands of medical men.

---

Illustrations of Dissections in a Series of Original Colored Plates the Size of Life Representing the Dissection of the Human Body. By George Viner Ellis and G. H. Ford, Esq. Reduced on a uniform scale and reproduced facsimile, expressly for Wood's Library of Standard Medical Authors. Vol. II. New York: Wm. Wood & Co. Wood's Library of Standard Medical Authors. 1882.

We have already noticed the first volume of this work, and what was said of it applies also to this. The colored plates in our opinion are well executed, and, while we have not had an opportunity to compare the copies with the original, we must say, taking them by themselves, that they are not a legitimate subject for adverse criticism. The two volumes will not be out of place in the annual series.

